# *Arabidopsis thaliana*: a powerful model organism to explore histone modifications and their upstream regulations

**DOI:** 10.1080/15592294.2023.2211362

**Published:** 2023-05-17

**Authors:** Yang Yu, Sihan Wang, Ziqin Wang, Renwei Gao, Joohyun Lee

**Affiliations:** Division of Natural and Applied Sciences, Duke Kunshan University, Kunshan, Jiangsu, China

**Keywords:** *Arabidopsis thaliana*, model organisms, histone modifications, epigenetics

## Abstract

Histones are subjected to extensive covalent modifications that affect inter-nucleosomal interactions as well as alter chromatin structure and DNA accessibility. Through switching the corresponding histone modifications, the level of transcription and diverse downstream biological processes can be regulated. Although animal systems are widely used in studying histone modifications, the signalling processes that occur outside the nucleus prior to histone modifications have not been well understood due to the limitations including non viable mutants, partial lethality, and infertility of survivors. Here, we review the benefits of using *Arabidopsis thaliana* as the model organism to study histone modifications and their upstream regulations. Similarities among histones and key histone modifiers such as the Polycomb group (PcG) and Trithorax group (TrxG) in *Drosophila,* Human, and Arabidopsis are examined. Furthermore, prolonged cold-induced vernalization system has been well-studied and revealed the relationship between the controllable environment input (duration of vernalization), its chromatin modifications of *FLOWERING LOCUS C* (*FLC*), following gene expression, and the corresponding phenotypes. Such evidence suggests that research on Arabidopsis can bring insights into incomplete signalling pathways outside of the histone box, which can be achieved through viable reverse genetic screenings based on the phenotypes instead of direct monitoring of histone modifications among individual mutants. The potential upstream regulators in Arabidopsis can provide cues or directions for animal research based on the similarities between them.

## Introduction

Eukaryotic nuclear DNA is packed into chromatin, a condensed structure whose basic unit is the nucleosomes [1]. The core of nucleosomes is composed of a histone octamer containing two copies of histone H2A, H2B, H3, and H4 proteins, and an approximately 146-base-pair DNA segment wrapped around it [2,3]. Other than providing structural support, histones protrude their highly basic side chains from the nucleosome, receiving extensive covalent modifications on the tails, affecting inter-nucleosomal interactions and thus altering the chromatin structure and DNA accessibility [4–6]. Various histone modifications can be recognized by effector modules, which then transduce signals for further chromatin structural or functional changes [7]. The status of histone modification can switch by ‘writers’ and ‘erasers,’ underlying the dynamics of the chromatin structure and the process of transcriptional initiation and elongation [5,8]. The restructuring and remodelling of chromatins result in controlling the level of transcription through the corresponding histone modifications [9,10]. These modifications transduce the inheritance of gene expression patterns without altering the underlying DNA sequences, but by adapting chromatin structures [11,12].

Histone modifications have attracted significant attention in biomedical research due to their involvement in cancer onset, neurological diseases, and embryonic development [13–15]. Because of the limitations of human research, studying the basic science of histone modifications in other organisms is an essential requirement. The ideal model organism for studying histone modifications should possess orthologous genes and the usual characteristics that make a good model system, including well-studied genomic features, the feasibility to maintain and reproduce in the lab settings with a short generation time, and the capacity to generate mutants [16]. Additionally, the model organism should enable researchers to interconnect multiple layers of genetic alterations with phenotypic changes to determine the role of these genes in fundamental biochemical processes [17–19]. Among animal models, *Drosophila* and mice are the most chosen model organisms for histone modification studies because of their well-studied genetic frameworks and the conserved molecular pathways that are relevant to human diseases [20,21]. However, there are challenges that impede a broader inspection of the histone modifications using these animal models. First, the knock-out of a key histone regulator frequently leads to embryonic deaths and/or infertility in the animal models, which limits the spectrum of the study [22–24]. As an alternative, gene knock-down is performed as one of the major approaches in animal models to examine the function of a gene through targeted reduction of its expression level [25–28]. The available knock-out mutants listed in major databases of *Drosophila* and mice cannot cover all epigenetic pathways. As an example, in the Mouse Genome Informatics (http://www.informatics.jax.org/) database, 189 epigenetics-related genes can be found. By matching the gene symbols with records in Mutant Mouse Resource & Research Centers (MMRRC), a national mutant mice repository, only 35 knock-out strains are found to be available [29]. The mutant availability of Arabidopsis enables comprehensive reverse genetic screenings of the upstream regulators in the signalling pathway using subsequent phenotypic changes as the indicator, which reflects the level of environmental inputs and corresponding switching of histone modifications [30]. The available mutants allow a high-throughput screening of phenotypes to study the upstream signalling, without direct monitoring of histone modification in each mutant line required at the initial genetic screening. Second, transgenerational inheritance of environment-induced histone modifications is still not consistently observed in animal models [31,32]. The complex and changeable information behind histone modifications may not be able to accurately flow from parents to offspring, leaving potential obstacles to the research across multiple generations. Also, studies on the topic including cancer, ageing, and drug development have addressed the significance of the upstream signalling pathways that convert the environmental stimuli to epigenetic changes [33–35]. Due to the limitation of mutant screening in animal models, the exploration of upstream regulators from the environment involved in signalling histone modification from the outside of the nucleus has been rarely accomplished, and the profile of upstream signalling pathways of histone modifications across development remained uncovered [36].

Various studies have shown that the mechanisms of histone modifications and their molecular components are highly conserved evolutionarily across higher eukaryotes including plants, suggesting that plants research has the potential to unravel epigenetic pathways that are yet not completely understood and complement research conducted by animal models [37–40]. According to The Arabidopsis Information Resource (TAIR), a comprehensive *Arabidopsis thaliana* information database, 131 genes are annotated with epigenetics-related functions and all of their corresponding knock-out mutants are available, which implies that they are not infertile or lethal [41]. Moreover, there are over 260,000 individual mutant lines of *Arabidopsis thaliana* generated via *Agrobacterium*-mediated T-DNA mutagenesis (SALK, SAIL, and GABI-KAT lines), covering almost all *Arabidopsis thaliana* genes [42–45]. These cost-effective and easy-to-handle random insertional mutants can be easily accessed in multiple *Arabidopsis thaliana* stock centres worldwide, such as Arabidopsis Biological Resource Center (ABRC) (https://abrc.osu.edu), Nottingham Arabidopsis Stock Centre (NASC) (https://arabidopsis.info) and GABI-Kat (https://www.gabi-kat.de). Additionally, plants are immobile and so are more plastic in post-embryonic development to react to changing environmental conditions [46], which then minimizes behavioural artefacts. Consequently, a robust inheritance of histone modifications is observed in *Arabidopsis*, making it a good candidate to investigate cues of epigenetic memory [38,47].

The research using plant models has contributed to understanding the process that upstream environment inputs alter histone modifications and gene expression patterns [48]. For example, the well-studied vernalization system, a temperature-sensing process by which exposure to the prolonged cold during winter, leads to an epigenetic switch that permits flowering in the spring, provides a quantitatively controllable experimental system for histone modifications. In such a case, environmental factors regulate the establishment and maintenance of epigenetic modifications, which eventually affect gene expressions and phenotypic changes [49,50]. *Arabidopsis thaliana* enables the study of cold-induced environmental epigenetics of *FLOWERING LOCUS C* (*FLC*) for the following reasons: (1) competence to flower via vernalization is determined by chromatin modifications of *FLC* expression, (2) cold exposure can be quantitatively controlled, and (3) mutants of chromatin control in vernalization process can be easily identified by monitoring flowering time. Polycomb Repressive Complex 2 (PRC2) and Trithorax group (TrxG) complexes are found in both plants and animals and confer the balancing of *FLC* expression via quantitative depositions of H3K27me3 and H3K4me3 on *FLC* chromatin. During cold exposure, *FLC* expression is gradually reduced via increased Histone 3 lysine 27 trimethylation (H3K27me3) deposition on *FLC* chromatin by PRC2, and the decrease of Histone 3 lysine 4 trimethylation (H3K4me3) deposition was also monitored [30,51–53]. After vernalization, *FLC* silencing is stably maintained even when the plant grows in warm conditions via PRC1. Because *FLC* expression in Arabidopsis is the only determinant to promote flowering in spring, the flowering time is correlated with the duration of cold treatment, deposition of H3K27me3 on *FLC* chromatin, and the level of *FLC* expression. This example demonstrates the potential of using Arabidopsis as a controllable system to explore mechanisms and components transducing signals from the environmental stimuli to the responses inside the nucleus.

## Similarity of histone proteins between animals and plants

Histone proteins are highly conserved in eukaryotes, with structural constraint likely resulting from necessary assembly into the histone octamers and functional constraint from compact binding on DNA [54–58]. Among the histone family members, histones H3 and H4 are the most evolutionarily conserved and they bind to the DNA terminal segments that enter or leave the nucleosome, playing a more outstanding role in chromatin formation regulation [55,59–61]. Based on the evolutionary analysis of histone H3 protein, a high level of sequential conservation is maintained among eukaryotes, and ancient genome duplication events led to functional histone variants like H3.1 and H3.3 [62–65]. H3.3 differs from H3.1 at only three or four amino acids across species [66–69]. The expression of H3.1 is mainly detected during the S phase, which is coupled with DNA synthesis during replication [70–73]. In contrast, H3.3 is expressed throughout the whole cell cycle, serving as the replacement variant for the DNA-synthesis-independent deposition pathway [74,75]. Histone H4 proteins are also highly conserved among higher eukaryotes, which can be explained that the H4 genes have experienced strong purifying selection and birth-and-death evolution [76]. In addition, despite the independent emergence of many histone variants across animals and plants, their amino acid sequences show a limited number of differences at approximately the same loci, implying a selective pressure in evolutionary history [77].

The sequence alignment of the N-terminal tails in *Arabidopsis thaliana* histone subunits H3.1, H3.3, and H4 with those of other species indicates a high level of conservation across organisms (Table 1 and Figure S1). As expected, *Arabidopsis thaliana* H3.1, H3.3, or H4 shows a high level of identity and similarity with the human H3.1 (98% identity and 100% similarity), H3.3 (98% identity and 100% similarity) or H4 (100% identity and 100% similarity). However, it is important to note that sequence similarities may not always represent the same function as shown by previous research which compares Arabidopsis *UVR8* receptor sequence with RCC1 protein family in animals [78].

**Table 1.** The identity and similarity of N-terminus tail in histone subunits H3.1, H3.3, and H4 of Arabidopsis thaliana across organisms.

	Histone 3.1		Histone 3.3		Histone 4	
Species	Identity	Similarity	Identity	Similarity	Identity	Similarity
*Caenorhabditis elegans*	N/A	N/A	100%	100%	100%	100%
*Canis lupus familiaris*	98%	100%	98%	100%	100%	100%
*Drosophila melanogaster*	N/A	N/A	98%	100%	98%	100%
*Homo sapiens*	98%	100%	98%	100%	100%	100%
*Macaca mulatta*	98%	100%	98%	100%	100%	100%
*Mus musculus*	98%	100%	98%	100%	100%	100%
*Rattus norvegicus*	98%	100%	98%	100%	100%	100%

The basic C-terminal domain (CTD) of histone proteins is critical for chromatin binding and the N-terminal tail of histones modulates nucleosome structure and function [79,80]. Covalent modifications on the N-terminal tail of histone, including methylation and acetylation, are found in mammals and plants [5,81,82]. Most organisms share the modifications in the same amino acids, including H3K36, H3K4, and H3K27, resulting in similar transcriptional controls [83,84]. Lys-to-Met (K-to-M) substitutions of a key amino acid of histone H3, as an example, showed dominant-negative inhibition in all humans, *Drosophila*, and Arabidopsis [77,85–88]. Studies using human embryonic kidney–293T (HEK293T) cells suggested that exogenously expressed histone H3 Lys-36 to Met (H3K36M) mutant protein resulted in a global reduction of endogenous H3K36 methylation [85,86,89,90]. In *Drosophila*, overexpression of H3K36M in the eye decreases the H3K36me2, suggesting its dominant-negative effect on lysine methyltransferases [91]. In Arabidopsis, overexpressed H3K36M also induces a global H3K36 hypomethylation in the same dominant-negative manner [85,92].

Another example is the histone H3.3, which is required for reprogramming events during development across species and is associated with genes that are actively transcribing in both animals and plants [93]. Histone H3.3 is encoded by two genes in *Drosophila* (*H3.3A* and *H3.3B*) and human (*H3f3a* and *H3f3b*), and the knock-out mutants of one of either genes show decreased viability, partial lethality or infertility of the survivors [69,94,95]. Knock-down studies in mice also showed that the complete depletion of H3.3 caused early embryonic lethality, suggesting the significance of H3.3 in animal development [96]. In Arabidopsis, *HTR4*, *HTR5*, and *HTR8* encode H3.3. Although the triple knockout is lethal, *HTR4-HTR5*-double-knockout-*HTR8*-RNAi lines could survive [93]. Research on this mutant found a reduced linker H1 deposition and thus increased gene body DNA methylation [93]. The viable H3.3 mutant indicates that Arabidopsis can contribute to understanding the underlying mechanisms of histone proteins by its various knock-out mutants. Such examples also support that histone research with plants will complement animal models in answering unresolved questions that can be difficult to address with animal models.

## Similarity of histone modifier between animals and plants: PcG and polycomb complexes

Other than homologies in histone proteins and modification types, the shared or similar modifying mechanisms and conserved modifiers between Arabidopsis and animal models are also of great importance. Polycomb group (PcG) proteins were originally identified in *Drosophila* as the repressors of homoeotic (*Hox*) genes during embryonic development [97]. PcG homologs were then identified in various species including multicellular plants, animals, and some unicellular organisms, which suggests that the emergence of PcG proteins may trace back to early in eukaryotic evolution [98,99]. Although sequence similarities are not always correlated to the functional similarity between species [100,101], inter-species protein homology searching is a tool to recognize cues for function prediction [102–104]. In higher eukaryotes, PcG proteins constitute a global gene silencing system that is essential for maintaining cell identity and regulating growth and development [105,106]. PcG proteins are assembled in several distinct transcriptional-repressive complexes, among which the two major groups are Polycomb repressive complex 1 (PRC1) and 2 (PCR2) [107]. PRC2 catalyses mono-, di- and trimethylations of lysine 27 of histone H3 (H3K27me1/2/3) and PRC1 functions to maintain the H3K27me3 established by PRC2 as well as introduce H2A lysine 119 ubiquitination (H2AK119ub1) [108–111].

**Figure 1.**
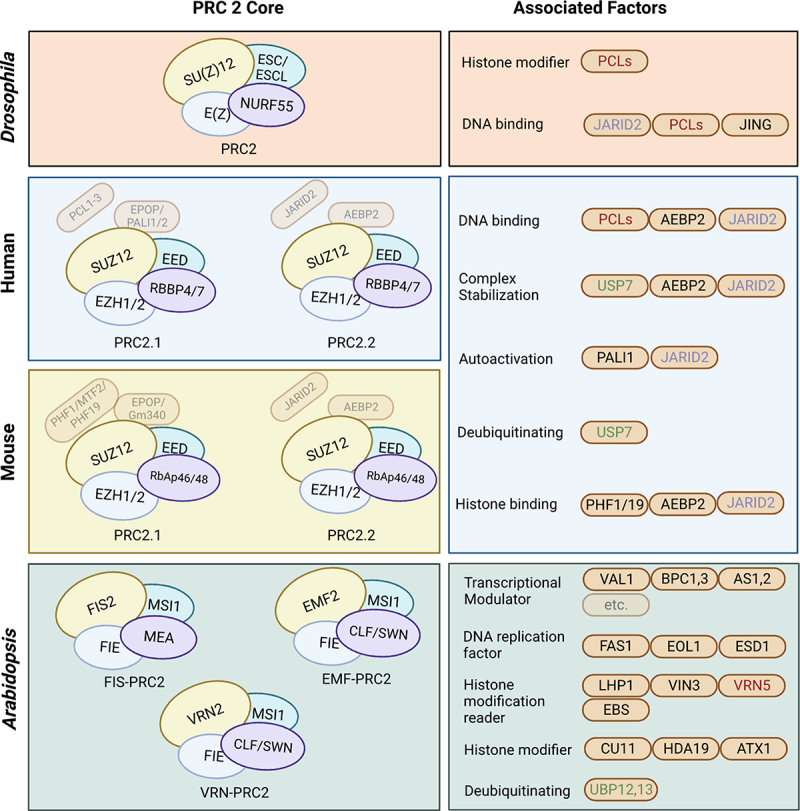
Comparison of PRC2 core components and their associated factors among *Drosophila*, human, mouse, and Arabidopsis. the interaction of PRC2 core components is represented among *Drosophila*, human, and Arabidopsis. The core components used the same colour between the organisms indicate corresponding orthologous proteins. The associated factors are classified by their functions, and their colour-coding indicates orthologous proteins between organisms.

PRC2 establishes H3K27 methylation in various organisms through the cooperation of the highly conserved core complex [112,113]. The *Drosophila* PRC2 core comprises four subunits: Enhancer of zeste (E(Z)), Extra sex comb (ESC/ESCL), Suppressor of zeste 12 (SU(Z)12), and NURF55 [114] (Figure 1). The former three subunits confer the histone methyltransferase activity and RBBP4/7 functions of PRC2 to bind to unmodified nucleosomes, which establish and maintain chromatin structures [115–117]. Correspondingly, functional and structural homologs of PRC2 core components in humans are E(Z) (EZH1/2), ESC/ESCL (EED), SU(Z)12 (SUZ12), and NURF55 (RBBP4/7) [118]. In mouse, the functional and structural homologs of PRC2 core components are E(Z) (EZH1/2), ESC/ESCL (EED), SU(Z)12 (SUZ12), and NURF55 (RbAp46/48) [119]. In Arabidopsis, homologous genes encoding the PRC2 core complex are also found, which include E(Z) (MEA, CLF, and SWN), ESC/ESCL (FIE), SU(Z)12 (FIS2, EMF2, and VRN2), and NURF55 (MSI1) [99], and these components are all orthologs with *Drosophila*, mice, and human, which are represented in the same colour of Figure 1. PRC2 mediates transcriptional repression via globally monitored H3K27me3 in *Drosophila* [114,120,121], mammals [122], and plants including Arabidopsis [123]. PRC2-mediated histone methylation requires targeted recruitment of PRC2 factors to the specific region of the chromatin [124,125]. Although distinct models are proposed to explain recruitment processes at a certain level, cross-species similarity is found in all key steps of each model. In *Drosophila*, PRC2 interacts with DNA-binding proteins to recognize and catalyse H3K27 methylation at Polycomb Response Elements (PREs) loci and DNA fragments with transcriptional factor (TF) binding site, with the latter contributes to the PRC2 recruitment [108,126]. Loci like PREs are also found in mammals and Arabidopsis with fixed-binding transcription factor families [127–131]. However, a PRE-KO experiment in *Drosophila* demonstrates that PREs may not be a necessary condition for H3K27me3 modification [132] and functional PREs are not globally identified in the genomes of mammals yet [133,134]. In mammals, PRC2 tends to bind unmethylated CG-rich CpG islands at the promoters of target genes [126,135,136]. Besides, many long noncoding RNAs (lncRNAs), including HOTAIR, Kcnq1ot1, and Braveheart, could recruit PRC2 through specific RNA-protein binding in mammals [137]. In Arabidopsis, lncRNAs such as COLDAIR [138], COOLAIR [139], and COLDWRAP [140] also participate in the recruitment of PRC2 to *FLC* chromatin. Besides, R-loop, a DNA-RNA hybrid structure, is also suggested to promote PRC2 recruitment in *Drosophila*, mammalian, and Arabidopsis through similar mechanisms [141–143].

PRC2 core complex can gain functional specificity by associating with partner proteins that can regulate PRC2 methyltransferase activity and/or its recruitment to the specific sites of the genome [126]. This gaining function via the associated factors mechanism was also conserved across species (Figure 1). In *Drosophila*, Polycomb-like (Pcl)’s binding to PRC2 is essential for high levels of H3K27me3 [144]. Pcl, Jarid2, and Jing also contribute to the recruitment of PRC2 to PRE [111]. In mammals, the N-terminal portion of SUZ12 interacts with various additional associated factors, leading to at least two different PRC2 assemblies, PRC2.1 (core assembly plus PCL1–3, EPOP, and PALI1/2) and PRC2.2 (core assembly plus AEBP2 and JARID2) with distinct biochemical properties [145]. Similarly, three PCR2 complexes, FIS-PRC2, EMF-PRC2, and VRN-PRC2, were found in Arabidopsis to control various developmental processes [146,147]. In human, associated factors including PCLs, AEBP2, and JARID2 play a role in DNA binding and complex recruitment; AEBP2 and JARID2 in complex stabilization; JARID2 and PALI1 in allosteric autoactivation; and PHF1/19, JARID2 and AEBP2 in binding to modified histones [83]. Similarly, in Arabidopsis, a complex protein–protein interaction network is formed through interactions among PRC2 core components and associated factors that share both functional and structural homology with counterparts found in other species. Those factors can be classified as transcriptional activators and repressors (VAL1, BPC1, BPC3, AS1, AS2, etc.), DNA replication factors (FAS1, ESD7, EOL1), histone modification readers (LHP1, VIN3, VRN5, SHL, EBS), histone modifiers (CU11, ATX1, HDA19), and ubiquitin C-terminal hydrolases [83,148]. Arabidopsis VIN3 and its homolog VRN5 are required in vernalization-induced histone modification and have multiple conserved domains with human PCL proteins that regulate the PRC2 recruitment and enzymatic activity. Arabidopsis shares multiple Jarid2 homologs (JMJ14, JMJ16, JMJ17) with animals [149]. Arabidopsis LHP1, a transcription modulator in PRC2 is a homolog of human HP1 that is considered exclusive in PRC1 that participates in transcriptional regulation. UBP12 and UBP13 that remove mono-ubiquitination of histone H2A in Arabidopsis show sequence similarity to human ubiquitin-specific protease USP7 [150–156].

PRC1 is less evolutionarily conserved between animals and plants because of the divergence events early in animal evolution [157]. The core subunits of canonical PRC1 (cPRC1) found in *Drosophila* are the E3 (Sce/dRING and Psc/Su(z)2) monoubiquitin ligase module and associated Pc, Ph, and SCM [150]. Ph and Pc are essential for higher-order chromatin folding of *Hox* clusters and Pc specifically binds to H3K27me3 [158,159]. SCM is a functional link and a key mediator between PRC1 and PRC2 in transcriptional silencing [160]. Most of the components of cPRC are missing in plants, but in Arabidopsis, three homologs of Psc (AtBMI1a, AtBMIb, and AtBMI1c) and two homologs of dRING (AtRING1a and AtRING1b) have been identified [146], which are a PRC1-like complex that incorporates some PRC1 homologs, but also plant-specific factors [147]. The previous model describes that the cPRC1 complex recognizes methylation of the histone by PRC2 and in turn maintains the trimethylation mediated by PRC2 and catalyses suppressive mono-ubiquitination. The same H3K27me3-governing function at major PcG targets, such as *FLC*, is executed by Arabidopsis LHP1, an H3K27me3-binding factor [161]. Evidence also indicates that the activity of PRC1 may be independent of PRC2 [162].

## Similarity of histone modifier and chromatin remodeler between animals and plants: TrxG and trithorax complexes

**Figure 2.**
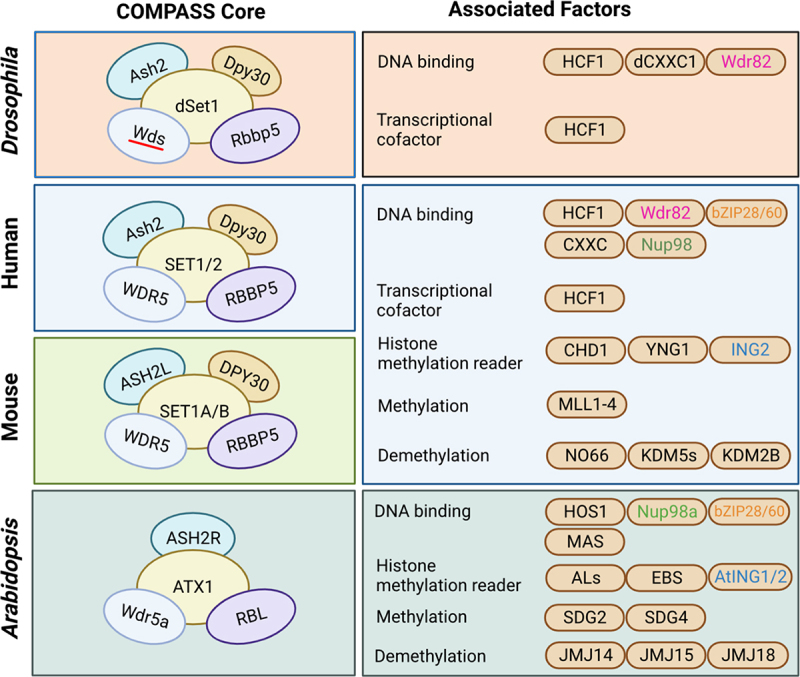
Comparison of COMPASS core components and their associated factors among *Drosophila*, mouse, human, and Arabidopsis. The interaction of COMPASS core components is represented among *Drosophila*, mouse, human, and Arabidopsis. The core components used the same colour between the organisms indicate corresponding orthologous proteins. The associated factors are classified by their functions, and their colour-coding indicates orthologous proteins between organisms.

Trithorax group (TrxG) proteins function to establish, maintain, and transmit the gene expression patterns mainly through methylating lysine 4 of histone H3 (H3K4) and remodelling nucleosomes [163,164]. TrxG is first identified in *Drosophila* as the transcription activator of *Hox* gene, which has the opposite to transcription-suppressive PcG proteins [165–167]. Similar to PcG, homologs of the TrxG were found in broader species, across yeast, animals, and plants [168–171], and they can be classified into three groups based on their functions [172]. The first group is the SET domain-containing factors that are involved in transcriptional activation, and more specifically, related to di- and tri-methylation of H3K4 [133,173,174]. Such SET-containing proteins are found in both animals and plants, such as SET1 and TRX in *Drosophila*, MLLs in humans, SET1A/B and MLLs in mice, and ATXs and SDGs in Arabidopsis [163,175,176] (Figure 2). Another group is the ATP-dependent chromatin-remodelling factors, such as BRM in *Drosophila*, BRG1 and hBRM in humans, and BRM and SYD in Arabidopsis [163,177,178]. Proteins from the third group can combine with certain DNA sequences, and it consists of some unclassified proteins from the former two groups and some histone modifiers and chromatin remodellers [163]. Collectively, TrxG proteins are assembled into multiprotein complexes to exert their functions as a whole complex [163]. A major group of TrxG complex includes COMPASS and COMPASS-like, which catalyses the trimethylation of the histone H3 lysine 4 (H3K4me3). Another group, represented by SWI/SNF, mediates ATP-dependent chromatin remodelling and nucleosome structure altering [172,179].

COMPASS complexes, also known as SET1A/B, mediate H3K4me3 as an activation mark at non-transcribing regions of the gene, including 5’ regions and promoters in animals and plants [180–182]. COMPASS has a core of four (in Arabidopsis) or five (in human, mice, and *Drosophila*) component modules (Figure 2), which is necessary for the complex assembly of COMPASS in both mammals and plants [183]. The COMPASS core is highly conserved in almost all higher eukaryotes [184]. In human, SET domain-containing protein SET-2/SET1 is a conserved methyltransferase [184,185], Ash2 is required for H3K4me3, DPY30 is required for H3K4me2 and H3K4me3, and RBBP5 and WDR5 are required for the assembly of COMPASS complex. The *Drosophila*’s homologs are SET1/2 (dSet), WDR5 (Wds), Ash2 (Ash2), RbBP5 (Rbbp5), and Dpy30 (Dpy30), respectively. In mice, they are SET1/2 (SET1A/B), WDR5 (WDR5), Ash2 (ASH2L), RbBP5 (RBBP5), and Dpy30 (DPY30) [186]. Arabidopsis retains four out of five homologs of the human COMPASS complex core components [182]. The homolog of human SET1/2, ASH2, and RbBP5 in Arabidopsis are ATX1, ASH2R, and RBL. The Arabidopsis homologs for human WDR5 are WDR5a and WDR5b. WDR5a binds to histone H3 tails and can form the core complex with ASH2R and RBL together [182], while WDR5b does not interact with RBL to form the structural platform of the COMPASS complex.

COMPASS also acquires certain functions by interacting with associate factors (Figure 2). In both animals and plants, several associated factors of the COMPASS complex are responsible for the writing, reading, and erasing of the histone methylation [187]. In mammals, MLL1–4 performs as the writer of histone methylation [187–190] with SDG2 and SDG4 acting as the writers in Arabidopsis [188,189]. The readers in Arabidopsis are ALs, AtING1,2, EBS, etc., whereas in mammals they are CHD1, YNG1, ING2, etc. [187,191,192]. NO66, KDM2B, and KDM5A/B/C/D are the erasers in animals, while JMJ14, JMJ15, and JMJ18 are the demethylases in Arabidopsis [187,193–196]. Some associated factors mediate the recruitment of the COMPASS component. For example, bZIP28 and bZIP60 can interact with WDR5 and Ash2 respectively in Arabidopsis, and with SWD3 and BRE2 in mammals, to direct the COMPASS complex to the promoters of unfolded protein response (UPR) genes [197,198]. In Arabidopsis, MAS can recruit COMPASS to MAF4 by interacting with WDR5a [199]. In mammals, Nup98, is a subset of nucleoporins that can recruit COMPASS complex to promoters to regulate H3K4me3 [200]. In humans, HCF1, CxxC, Nup98, Wdr82, bZIP28, and bZIP60 facilitate DNA binding, whose functional homologs in *Drosophila* are HCF1, dCxxC1, and Wdr82 while in Arabidopsis are HOS1, MAS bZIP28 and bZIP60.

SWI/SNF functions to mediate ATP-dependent chromatin remodelling, one of the basic mechanisms required for the compaction of the nucleosome [201–203]. SWI/SNF complexes were originally discovered in *Saccharomyces cerevisiae*, and similar complexes were characterized later in *Drosophila* [204,205]. Conserved among eukaryotes, SWI/SNF complexes utilize the energy from ATP hydrolysis to slide the DNA around the nucleosome, reposition the nucleosome and allow transcriptional apparatus to be recruited to the DNA [172]. The catalytic core subunit of the complex shows cross-species conservation in mammals and Arabidopsis. BRM/BRG1 in mammals is homologous to SWI/SNF in yeast [206]. In human, one catalytic subunit, BRG1 or BRM, associates with multiple non-catalytic subunits, forming three complexes: canonical BAF, Polybromo-associated BAF, and noncanonical BAF [207]. BAFs are important in multiple cellular processes, and their dysfunctions are the risk factors for cancer [203,208]. Although there is no plant endogenous SWI/SNF complex, two homologs to SWI/SNF catalytic subunits, BRM and SYD, are found in Arabidopsis and they participate in the development and phytohormone responses [209].

Initially discovered in *Drosophila*, PcG proteins collectively act as repressors of *Hox* genes, while the gene activation is mediated by TrxG proteins [165]. Later work has broadened the spectrum of regulatory elements that can recruit PcG and trxG factors, which are named PcG and trxG response elements (PREs and TREs) [210–212]. Trx associates with CREB-binding protein (CBP), mediates H3K27 acetylation, and prevents H3K27me3 at Polycomb target genes [213]. TrxG protein Ash1 antagonizes PcG-mediated silencing via H3K36 di-methylation at *HOX* genes [214,215]. On contrary, Pc inhibits the H3K27 acetylation by blocking the acetyltransferase activity of CBP and consequently favours H3K27 methylation [216]. With the opposite regulatory functions, the TrxG and PcG complexes compete dynamically, maintaining the balance between genetic repression and activation in various organisms. In Arabidopsis, PcG protein CLF acts as a transcriptional repressor of the *AGAMOUS* (*AG*) gene through H3K27me3 while TrxG protein ATX1 acts to keep high-level *AG* transcription, suggesting the antagonist function of ATX1 [217].

## Why Arabidopsis: open questions from environmental signal to histone box

More recent studies on the epigenetic components of cellular memory and plasticity have revealed that control of chromatin status is not limited to the modifications performed by the multiple complexes mentioned in the previous section. The chromatin structure is also shaped by the histone sliding and the incorporation of histone variants (e.g., H2A.Z) mediated by four subfamilies of ATP-dependent chromatin-remodelling enzymes: imitation switch (ISWI), chromodomain helicase DNA-binding (CHD), SWI/SNF, and INO80 [218,219]. Moreover, the histone modifications and DNA methylation are found to be interdependent processes, which together determine chromatin status [220,221]. Although histone modifications are highly conserved in the evolution of eukaryotes, DNA methylation has unique mechanisms observed in mammals and plants [222,223]. Mammalian genomes show particularly high CG methylation levels (70%–80% in general) [222]. CG methylation is established through DNA methyltransferases 3 alpha (DNMT3A) and DNMT3B and is maintained by DNMT1 upon replication [224]. CG methylation is implicated in the repressed gene expression in the promoter region but is associated with actively transcribed gene bodies [224]. In Arabidopsis, DNA methylation occurs in three cytosine sequence contexts: CG, CHG, and CHH (H=A, T or C). CG methylation is maintained by METHYLTRANSFERASE 1 (MET1), an orthologue of the mammalian DNMT1 [225]. CHG methylation and asymmetric CHH methylation are site-specifically methylated by CHROMOMETHYLASE 2 (CMT2) and METHYLTRANSFERASE 2 (DRM2) [226]. Gene body methylation is most frequent in constitutively and moderately expressed genes but not in genes with variable expression [227,228]. These findings indicate that there are multiple species-specific mechanisms evolved that intertwined together to control the chromatin status within the nucleus. Meanwhile, outside the nucleus, the signalling pathway connecting environmental stimuli and the intracellular histone modifications has received more attention in recent years, becoming a significant factor in understanding the field of biology, disease, and evolution [229–231]. However, how environmental signals are transferred from the plasma membrane to the nucleus has been still uncovered. Although there have been efforts to identify the upstream regulators outside the ‘histone box’ using large-scale data analyses, grossly perturbed epigenomes in each mutant make it hard to identify if the change of histone modifications compared to the wild type is direct effects [232].

Identification of the upstream regulatory components using mutant can be challenging in some animal models due to lethality and/or sterility. For example, loss-of-function mutations on genes encoding Polycomb proteins frequently cause lethality in mammalian development [233,234]. Homozygous mutations on EZH2, EED, and SUZ12, the core components of the PRC2 complex, lead to early embryonic lethality in mice, and mutants in H3K4 demethylase or methyltransferase produce initially healthy animals which give birth to an increasing percentage of sterile offspring after each generation [232,235,236]. Furthermore, controlling environmental input to establish a quantitative system in animal models and the corresponding phenotype caused by the same input would be challenging.

**Figure 3.**
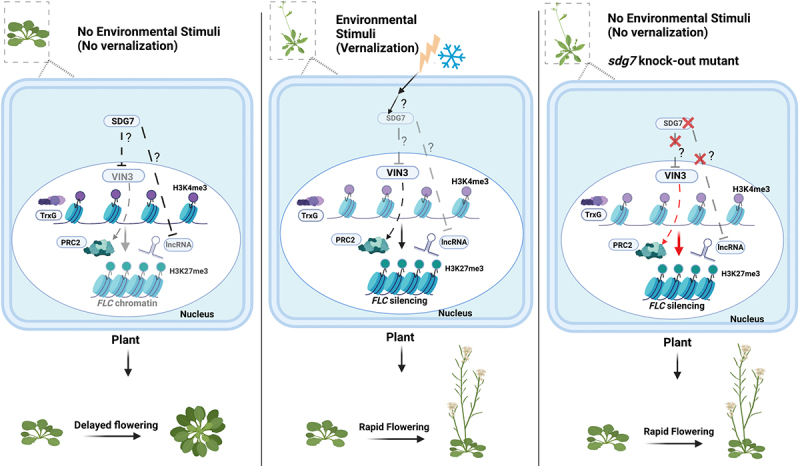
Vernalization pathway without or with prolonged cold exposure as an environmental stimulus in Arabidopsis. Quantitative correlations between the duration of cold exposure and the histone modifications results in changes of *FLC* chromatin status and its expression. Because the flowering time in vernalization pathway depends on *FLC* expression, the flowering time is switched based on the quantitative environmental input (left and middle panel). The *sdg7* mutant is unable to suppress the expression of the PRC2 component VIN3, as well as the lncRNAs COOLAIR and COLDAIR. This results in a decrease in *FLC* expression via an increase in H3K27me3, leading to rapid flowering without cold exposure (right panel).

Considering these aspects, *Arabidopsis thaliana* is a powerful model to identify upstream regulators of histone modifications. Moreover, the vernalization system in Arabidopsis brings advantages to reveal the relationship between the controllable environment input (duration of vernalization) correlate phenotypic change, chromatin modifications, and following gene expression. *FLC* locus is the key to conferring the cold-induced chromatin modification via the balance of the chromatin modification between PRC2 and TrxG, resulting in corresponding flowering time phenotypes [122,237,238]. Before cold exposure, the FRIGIDA (FRI) complex activates *FLC* together with the deposition of H3K4me3 mediated by TrxG, leading to the consequent suppression of flowering and delayed flowering time [239–241] (Figure 3, left panel). During cold exposure, the expression of a cold-specific PRC2 component, *VIN3*, is gradually induced, and *FLC* expression is gradually reduced via increased H3K27me3 deposition on *FLC* chromatin. After vernalization, regardless of *FLC* activation by FRI, PRC2-mediated *FLC* silencing is stably maintained even when the plant grows in warm conditions because of the function of PRC1 [242] (Figure 3, middle panel). The *vin3* mutant is vernalization insensitive, resulting in delayed flowering after vernalization [243]. In addition, three long noncoding RNAs (lncRNAs) generated from *FLC*, *COOLAIR* [138], *COLDAIR* [244], and *COLDWRAP* [140] are up-regulated during vernalization. VIN3 and three non-coding RNAs, COLDAIR, COOLAIR, and COLDWRAP have essential roles in the PRC2-mediated silencing of *FLC*. Interestingly, the plant homeodomain (PHD) finger domain of VIN3 is found to be critical for PRC2 recruitment in human cells [155,245]. Human polycomb-like (PCL) proteins, hPCL1, hPCL2, and hPCL3, contain plant homeodomain (PHD) finger domains, and participate in PRC2 recruitment to the target locus for the increase of H3K27me3 [245–248]. A human H3K27me3-methyltransferase, EZH2, binds to PHD finger domain of hPCL1 (PHF1) and methylates its target genes [155,245]. Therefore, VIN3 in the PRC2 complex is highly likely to have a homologous function to PCLs in human.

**Figure 4.**
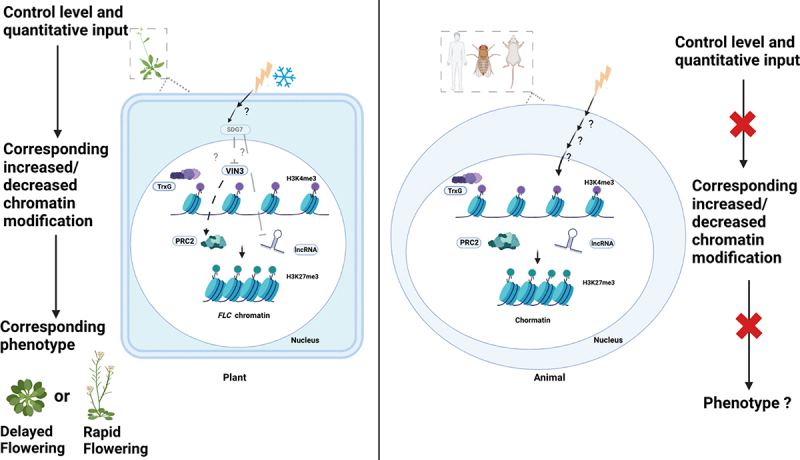
Comparison of regulations of the histone modification pathway between plants and animals in three aspects: environmental input, corresponding histone modifications, and resulting phenotypic changes.

In a previous genetic screening, multiple upstream regulators outside the histone box in Arabidopsis were identified [249]. *VIN3* is not expressed without cold exposure, thus the authors performed mutagenesis to isolate mutants that induce the expression of *VIN3* without vernalization using *VIN3-GUS* transgenic reporter lines treated ethyl methane sulphonate for random mutations [249]. VIN3 is a PRC2 component that suppresses *FLC* expression with cold exposure, the early flowering mutants with less expression of *FLC* are strong candidates for negative regulators of VIN3 without vernalization. If the candidates negatively regulate *VIN3*, the *vin3*/*candidate* double mutant should show the *vin3* mutant phenotype (delayed flowering after vernalization). Therefore, a mutant of the upstream negative regulator of *VIN3* should exhibit: (1) rapid induction of the *VIN3-GUS* reporter and thus native *VIN3*, (2) rapid flowering without cold exposure depending on the chromatin status of *FLC*, and (3) vernalization-insensitive flowering with the loss of *VIN3* in a double mutant. This experiment identified a mutant that meets the three criteria, the causative locus encodes a methyltransferase, SET DOMAIN GROUP7 (SDG7), and the mutant has a missense mutation in a highly conserved proline residue (P199L) in SET domain-containing proteins (*sdg7–1*) [249]. Furthermore, lncRNAs, COOLAIR, and COLDAIR were also induced in *sdg7* mutants without cold exposure. Increased deposition of H3K27me3 on *FLC* chromatin, resulting in decreased *FLC* expression, suggesting that SDG7 is an upstream regulator of VIN3 (Figure 3, right panel). Interestingly, SDG7 does not have a nuclear localization signal, and is not targeted at the nucleus [249,250]. Lys methylation activity of SDG7 was detected *in vitro* with an aquaporin substrate targeted to the plasma membrane [250], but no histone methyltransferase activity was detected *in vitro*. These data suggest that SDG7 is involved in the vernalization via Lys methylation of extranuclear proteins as a signalling process to control PRC2-mediated *FLC* silencing. Although Lys methylation of extranuclear proteins is widespread, its biological significance is unclear [251–253]. Arabidopsis SDG7 is an example of a potential upstream regulator of the PRC2 outside of the histone box (Figure 4, left panel), suggesting that Arabidopsis plant can be a model to identify upstream regulators from the environment to the nucleus using the controllable vernalization system. In plants, the quantitatively controlled input level tightly associates with the chromatin modification change that is revealed by the corresponding phenotypes, whereas in animals, the lack of such association hinders such phenotypic screening (Figure 4, right panel).

## Discussion

The involvements of epigenetic regulation, especially histone modifications in the response to environmental changes, have been revealed, but there are missing pieces that demonstrate how the extracellular signals are transduced into the nucleus. *Arabidopsis thaliana* can serve as exceptional experimental subjects to study the components of epigenetic signalling induced by environmental cues. It will also be a powerful model that opens up more opportunities for the understanding of histone modifications, especially to understand how upstream regulators located outside of the nucleus work to transduce environmental signals. Because of the high similarity of histone proteins, chromatin modification modules, the key protein complexes and associated components in animals and plants, the upstream signalling and its components could be conserved at the molecular level. In addition, findings in animal models have already provided numerous key inspirations guiding the studies in plant, i.e., the discovery of Polycomb and Trithorax proteins which are first identified in *Drosophila* [123]. The findings in *Arabidopsis thaliana*, vice versa, have great potential to provide cues for animal studies. Arabidopsis has made a significant contribution to the field of small RNA and related epigenetic mechanisms, such as the finding of the Argonaute gene and RNA-directed DNA methylation [254]. The chromatin remodelling factor DECREASE IN DNA METHYLATION 1 (DDM1) was first discovered in Arabidopsis mutant screenings and later mammalian ortholog Lsh was found with the same effect in centromeric methylation [254–256]. These efforts suggest that using Arabidopsis as a model organism for epigenetic studies can also make a contribution to research related to human health. Epigenetic switches of mammalian stem cells have been extensively studied. However, stem cell research using plants can be beneficial as an extension of mammalian research. The animal and plant kingdoms have diverged since 16 billion years ago, and the only conserved protein that specifically participated in the stem cell function among these two kingdoms is the retinoblastoma-related (RBR) protein [257,258]. Interestingly, their epigenetic modifying patterns sustaining the pluripotency of stem cells are dramatically parallel [259]. These patterns include the alteration of nucleosome conditions, histone acetylation or deacetylation, H3K27 or H3K4 methylation, chromatin compaction, and chromatin assembly or disassembly, where corresponding proteins and regulators are functioning following conserved general patterns in the epigenetic regulations of stem cells [259]. Investigations into stem cell reprogramming properties in plants and animals have also revealed the high conservation in their core regulatory logic and general machinery [257,258]. In addition, plant stem cells are more dependent on cell-to-cell communication, which is consistent with their need for receiving signals from the environment [258]. Combining the fact that differentiation and dedifferentiation naturally occur throughout their life span in plant stem cells, it can be a beneficial experimental subject to investigate the basic mechanisms of pluripotency restoration of cells [257,260].

The ease of cultivation provides another reason for applying plants as the model organisms for epigenetic research such as the short generation time of fewer than 2 months, the smaller size that makes it easier to handle larger scale samples in growth facilities, and the potential to maintain epigenomes between generations on account of the self-pollinations [261]. Furthermore, the small pan-genome size (~135Mb) with a significantly low content of interspersed repetitive DNA sequences may minimize redundancy issues at a genetic level [262–264]. Easier seed storage is also beneficial compared to the maintenance of mammalian offsprings, which require extensive care to be kept alive and healthy under genetic mutation or experimental treatments.

## Supplementary Material

Supplemental Material

## Data Availability

histone sequences used in this study are available at the UniProt (https://www.uniprot.org/) [265] and NCBI RefSeq (https://www.ncbi.nlm.nih.gov/refseq/) [266]. Accession numbers are listed in Supplement Table 1.
