# Supplementary material for: Arabidopsis thaliana: a powerful model organism to explore histone modifications and their upstream regulations
Source: Epigenetics. 2023 May 17;18(1):2211362. doi: 10.1080/15592294.2023.2211362 (PMC10193919; doi:10.1080/15592294.2023.2211362)
Supplement: Supplemental Material [file KEPI_A_2211362_SM2778.zip › Supplementary files/Supplementary figure and table.docx]

**Supplementary Figure S1. Alignments of N-terminus tail in histone subunits H3.1, H3.3, and H4 of *Arabidopsis thaliana* across organisms.** The alignment result of N-terminus tail in histone subunits H3.1(a), H3.3 (b), and H4 (c) across selected species are displayed. can be found in most of the species. For H3.1, *Arabidopsis thaliana* has one mismatch at position 42 (Tyrosine to Phenylalanine; both aromatic amino acids) compared to the other species, while for H3.3, *A. thaliana* and *C. elegans* have one mismatch at position 32 (Serine to Threonine; both hydroxylic amino acids) compared to the other species. For H4 the N-terminus is completely conserved in selected species except for Drosophila, which changes the Serine at position 2 to Threonine.

**Supplementary Table S1.** Data source of histone H3.1, H3.3, and H4 sequences in 8 species. NA: not available.

| **Taxa** | **Protein** | **Database** | **Accession Number** |
| --- | --- | --- | --- |
| *Arabidopsis thaliana* | H3.1 | UNIPROT | [P59226](https://www.uniprot.org/uniprotkb/P59226) |
|  | H3.3 | UNIPROT | [Q9LR02](https://www.uniprot.org/uniprotkb/Q9LR02) |
|  | H4 | UNIPROT | [P59259](https://www.uniprot.org/uniprotkb/P59259) |
| *Rattus norvegicus* | H3.1 | UNIPROT | [Q6LED0](https://www.uniprot.org/uniprotkb/Q6LED0) |
|  | H3.3 | UNIPROT | [P84245](https://www.uniprot.org/uniprotkb/P84245) |
|  | H4 | UNIPROT | [P62804](https://www.uniprot.org/uniprotkb/P62804) |
| *Macaca mulatta* | H3.1 | NCBI | [NP_001361473.1](https://www.ncbi.nlm.nih.gov/protein/NP_001361473.1) |
|  | H3.3 | NCBI | [XP_014970664.1](https://www.ncbi.nlm.nih.gov/protein/XP_014970664.1) |
|  | H4 | UNIPROT | [F7HAP8](https://www.uniprot.org/uniprotkb/F7HAP8) |
| *Homo sapiens* | H3.1 | UNIPROT | [P68431](https://www.uniprot.org/uniprotkb/P68431) |
|  | H3.3 | UNIPROT | [P84243](https://www.uniprot.org/uniprotkb/P84243) |
|  | H4 | UNIPROT | [P62805](https://www.uniprot.org/uniprotkb/P62805) |
| *Canis lupus familiaris* | H3.1 | NCBI | [XP_038302472.1](https://www.ncbi.nlm.nih.gov/protein/XP_038302472.1) |
|  | H3.3 | NCBI | [XP_038527592.1](https://www.ncbi.nlm.nih.gov/protein/XP_038527592.1) |
|  | H4 | UNIPROT | [F2Z4N2](https://www.uniprot.org/uniprotkb/F2Z4N2) |
| *Mus musculus* | H3.1 | UNIPROT | [P68433](https://www.uniprot.org/uniprotkb/P68433) |
|  | H3.3 | UNIPROT | [P84244](https://www.uniprot.org/uniprotkb/P84244) |
|  | H4 | UNIPROT | [P62806](https://www.uniprot.org/uniprotkb/P62806) |
| *Drosophila melanogaster* | H3.1 | NA | NA |
|  | H3.3 | UNIPROT | [P84250](https://www.uniprot.org/uniprotkb/P84250) |
|  | H4 | UNIPROT | [P84040](https://www.uniprot.org/uniprotkb/P84040) |
| *Caenorhabditis elegans* | H3.1 | NA | NA |
|  | H3.3 | UNIPROT | [Q10453](https://www.uniprot.org/uniprotkb/Q10453) |
|  | H4 | UNIPROT | [P62784](https://www.uniprot.org/uniprotkb/P62784) |
