# Supplementary figures and images for: Arabidopsis thaliana: a powerful model organism to explore histone modifications and their upstream regulations
Source: Epigenetics. 2023 May 17;18(1):2211362. doi: 10.1080/15592294.2023.2211362 (PMC10193919; doi:10.1080/15592294.2023.2211362)

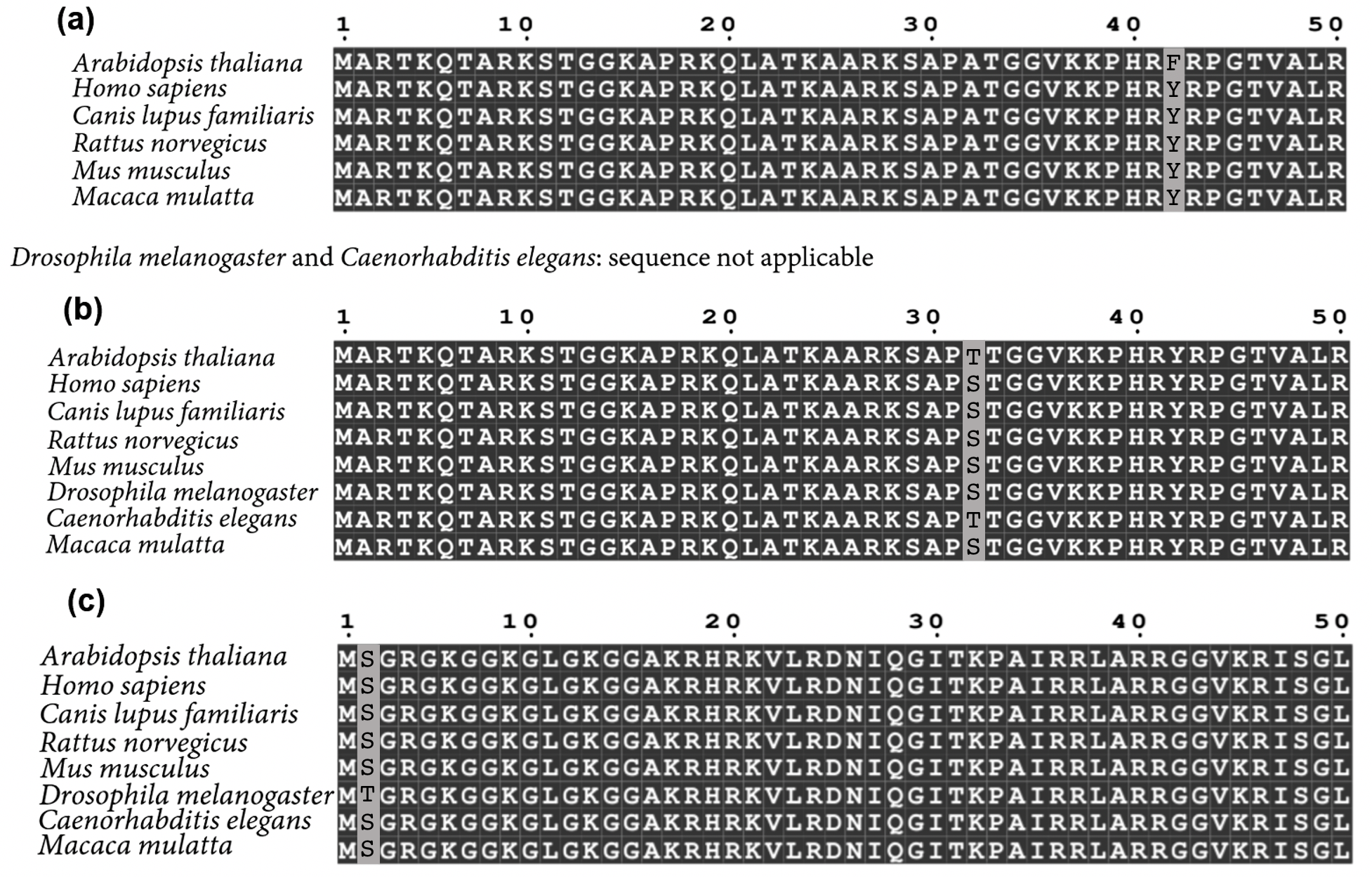

Supplement: Supplemental Material [file KEPI_A_2211362_SM2778.zip › Supplementary files/Fig S1.tif]
